# Effects of d- and l-limonene on the pregnant rat myometrium *in vitro*

**DOI:** 10.3325/cmj.2015.56.431

**Published:** 2015-10

**Authors:** Judit Hajagos-Tóth, Ágnes Hódi, Adrienn B. Seres, Róbert Gáspár

**Affiliations:** Department of Pharmacodynamics and Biopharmacy, Faculty of Pharmacy, University of Szeged, Szeged, Hungary

## Abstract

**Aim:**

To study the effects of d- and l-limonene on pregnant rat myometrial contractility *in vitro*, and investigate how these effects are modified by other agents. D- and l-limonene (10^−13^-10^−8^ M) caused myometrial contraction in a dose-dependent manner.

**Methods:**

Contractions of uterine rings from 22-day-pregnant rats were measured in an organ bath in the presence of d- or l-limonene (10^−13^-10^−8^ M) and nifedipine (10^−8^ M), tetraethyl-ammonium (10^−3^ M), theophylline (10^−5^ M), or paxilline (10^−5^ M). Uterine cyclic adenosine monophosphate (cAMP) level was detected by enzyme immunoassay. Oxidative damage was induced by methylglyoxal (3 × 10^−2^ M) and the alteration was measured via noradrenaline (1 × 10^−9^ to 3 × 10^−5^ M) -induced contractions.

**Results:**

Pre-treatment with nifedipine (10^−8^ M), tetraethylammonium (10^−3^ M), and theophylline (10^−5^ M) attenuated the contracting effect of d- and l-limonene, while in the presence of paxilline (10^−5^ M) d- and l-limonene were ineffective. The two enantiomers decreased the myometrial cAMP level, but after paxilline pretreatment the cAMP level was not altered compared with the control value. Additionally, l-limonene (10^−6^ M) diminished consequences of oxidative damage caused by methylglyoxal (3 × 10^−2^ M) on contractility, whereas d-limonene was ineffective.

**Conclusion:**

Our findings suggest that l-limonene has an antioxidant effect and that both d-and l-limonene cause myometrial contraction through activation of the A_2A_ receptor and opening of the voltage-gated Ca^2+^ channel. It is possible that limonene-containing products increase the pregnant uterus contractility and their use should be avoided during pregnancy.

Over the past few decades there has been a significant increase in the usage of self-prescribed complementary and alternative medication during pregnancy, such as herbal remedies, relaxation therapies, and aromatherapy (1-3). These products are mistakenly perceived to be safe and are mostly used in treating anxiety, insomnia, fatigue, back pain, constipation, and heartburn (1-5). For example, almond oil is widely believed to be a good nutrient for the developing fetus, however it has been proven to induce preterm birth (3). Since there is a lack of evidence on the safety of herbal medications and essential oils use during pregnancy, more information on this issue should be obtained.

Limonene is a chiral molecule classified as a monoterpene and occurring as two optical enantiomers: d-limonene or [R(+)-enantiomer] and l-limonene or [S(-)-enantiomer] (6). It is mostly present in plants in its d-enantiomeric form or, to a lesser extent, as a racemic mixture (7). Limonene is a major component of several plant essential oils, including orange, lemon, mandarine, lime, and grapefruit. Because of its pleasant lemon-like sweet fragrance it is widely used as an additive to perfumes, soaps, foods, chewing gums, and beverages (8). D-limonene is also used therapeutically to dissolve cholesterol-containing bilestones (9) and relieve heartburn (10). It is rapidly and almost completely absorbed from the gastrointestinal tract (11). D-limonene has low toxicity and does not have any mutagenic, carcinogenic, or nephrotoxic risk to humans (12). Furthermore, it has a chemotherapeutic (13), anti-inflammatory (14), anxiolytic (15), antinociceptive (16), and potent antioxidant effects (17). It modifies the myometrial relaxing effect of terbutaline and decreases its cervical resistance-enhancing effect, however little is known about its mechanism of action (18). Some monoterpenoids have known mechanisms of action: eg, camphor and borneol are non-competitive inhibitors of neural acetylcholine receptors (19) and thymol inhibits Ca^2+^ and K^+^ channels in human ventricular cardiomyocytes (20). Monoterpenes induce vascular smooth muscle relaxation in rat mesenteric arteries through activation of large conductance Ca^2+^-activated potassium (BK_Ca_) channels and inhibition of the L-type Ca^2+^ channels (21), and relaxation in isolated rabbit ileum through NO pathway and inhibition of L-type Ca^2+^ channels (22). Eucalyptol induces relaxation in rat and guinea-pig airway smooth muscle through non-specific mechanisms (23).

Since no experiments have been carried out to investigate the effect of limonene on myometrial smooth muscle, our primary aim was to study the effects of limonene on pregnant Sprague-Dawley rat myometrium on the last day of pregnancy *in vitro*. Since intracellular Ca^2+^ plays an important role in the mechanism of action of monoterpenoids (21-23), our second aim was to investigate how the myometrial effect of limonene can be modified in the presence of the L-type Ca^2+^ channel blocker nifedipine, selective BK_Ca_ channel blocker paxilline, non-selective potassium channel blocker tetraethyl-ammonium (TEA), and non-selective adenosine receptor antagonist theophylline.

## Materials and methods

All experiments were carried out with the approval of the Hungarian Ethics Committee for Animal Research (registration number: IV/198/2013). The animals were treated in accordance with the European Communities Council Directives (86/609/ECC) and the Hungarian Act for the Protection of Animals in Research (XXVIII. tv. 32.§).

### Mating of the animals

In each experiment, mature female (180-200 g) (n = 8) and male (240-260 g) (n = 3) Sprague-Dawley rats were mated in a mating cage. The cage has a time-controlled movable metal door separating male from female animals. Since rats are usually active at night, the separating door was opened before dawn. Within 4-5 hours after the possible mating, vaginal smears were taken and a sperm search was performed under a microscope at a 1200 × magnification. Female rats with positive smear and those in whom the smear was not taken due to vaginal sperm plug were regarded as first-day pregnant animals.

### Uterus preparation

Uteri were removed from rats (n = 8 in each experiment) (250-350 g) on the 22nd day of pregnancy. 5 mm-long muscle rings were sliced from uterine horns and mounted vertically in an organ bath containing 10 mL de Jongh solution (composition: 137 mM NaCl, 3 mM KCl, 1 mM CaCl_2_, 1 mM MgCl_2_, 12 mM NaHCO_3_, 4 mM NaH_2_PO_4_, 6 mM glucose, pH = 7.4). The organ bath was maintained at 37°C, and carbogen (95% O_2_ + 5% CO_2_) was bubbled through it. After mounting, the rings were equilibrated for about 1 h before the experiments were conducted, with a solution change every 15 min. The tension of myometrial rings was set at about 1.25 g. The tension of myometrial rings was measured with a gauge transducer (SG-02; Experimetria Ltd, Budapest, Hungary) and recorded with a SPEL Advanced ISOSYS Data Acquisition System (Experimetria Ltd, Budapest, Hungary).

### D- and l-limonene contractility studies

In each experiment, cumulative dose-response curves were constructed in the presence of d- and l-limonene (10^−13^-10^−8^ M) (Sigma-Aldrich, Budapest, Hungary). After each concentration of limonene was added, recording was performed for 300 s.

### Combination of d- and l-limonene with nifedipine, paxilline, TEA, and theophylline

After pre-treatment with nifedipine (5 minutes) (10^−8^ M), paxilline (10 minutes) (10^−5^ M), TEA (10 minutes) (10^−3^ M), and theophylline (10 minutes) (10^−5^ M) (all from Sigma-Aldrich), cumulative dose-response curves were constructed in the presence of d- and l-limonene (10^−13^-10^−8^ M). The incubation period was chosen based on the data from our previous studies (24,25).

### Methylglyoxal (MGO) studies

N-acetyl-cysteine (NAC) has already been demonstrated to reduce the inhibitory effect of MGO on rat mesenteric artery *in vitro* (26). For comparison, we examined the effect of NAC (10^−7^ M) and another well-known antioxidant, tocopherol (10^−7^ M) on MGO-treated myometrium. After 30 minutes of pre-treatment with MGO (3 × 10^−2^ M), l- and d-limonene (10^−6^ M), tocopherol (10^−7^ M), and NAC (10^−7^ M) (Sigma-Aldrich), contractions were elicited with noradrenaline (NA) (1 × 10^−9^ to 3 × 10^−5^ M) and cumulative dose-response curves were constructed in each experiment in the presence of propranolol (10^−5^ M) (Sigma-Aldrich) in order to avoid β-adrenergic action. After each concentration of NA was added, recording was performed for 300 s.

### Measurement of uterine cyclic adenosine monophosphate (cAMP) accumulation

Uterine cAMP accumulation was measured with a commercial cAMP Enzyme Immunoassay Kit (Sigma-Aldrich, Budapest, Hungary). Uterine tissue samples were incubated in an organ bath (10 mL) with isobuthylmethylxantine (IBMX) (10^−3^ M) and paxilline (10^−5^ M) for 10 minutes, after which d- and l-limonene (10^−13^, 10^−9^ M) were added for another 3 minutes. At the end of limonene incubation, forskolin (10^−5^ M) was added for another 10 min. After stimulation, the samples were immediately frozen in liquid nitrogen and stored until the cAMP extraction (27). Frozen tissue samples were then ground, weighed, homogenized in 10 volumes of ice-cold 5% trichloroacetic acid, and centrifuged at 1000 g for 10 min. The supernatants were extracted with 3 volumes of water-saturated diethyl ether. After drying, the extracts were stored at -70°C until the cAMP assay.

### Statistical analysis

All variables are presented as mean ± standard deviation. Differences in the contractile effect of d- and l-limonene were analyzed using the unpaired *t* test. Differences in the contractile effect of NA in the MGO experiments were analyzed using one-way ANOVA tests with the Dunnett's multiple comparison test. Concentration-response curves were fitted, areas under the curve (AUC) determined, and maximal inhibitory effects (Emax) calculated with the Prism 4.0 software (Graphpad Software Inc. San Diego, CA, USA). Differences between the control cAMP and other groups were analyzed using one-way ANOVA tests with the Dunnett's multiple comparison test. The value of *P* < 0.050 was considered statistically significant.

## Results

### Effects of limonene on pregnant rat myometrium

**Figure 1:**
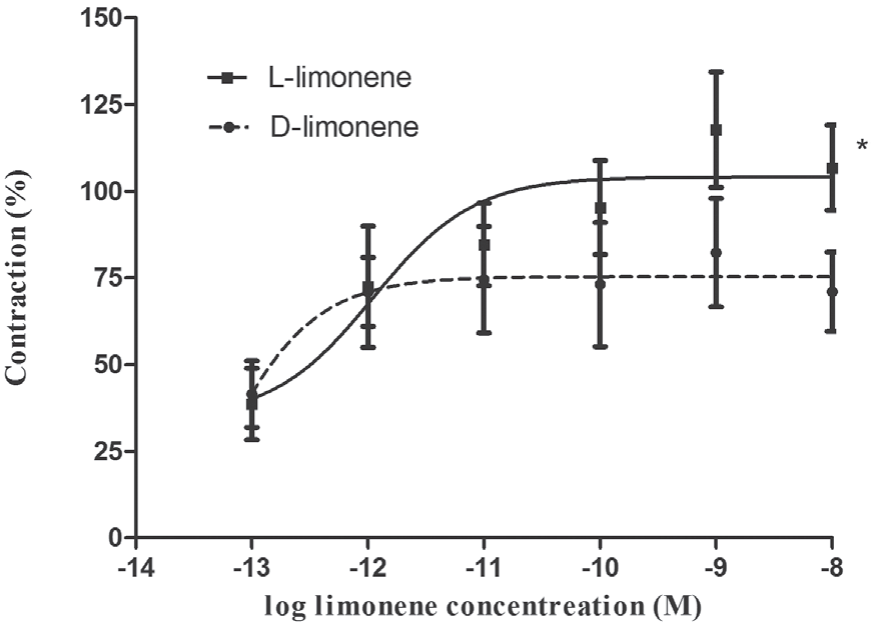
The effect of d- and d-limonene (10^−13^-10^−8^ M) on uterine contraction. Change in contraction intensity was calculated using the areas under the curve and expressed in percent ± standard deviation (unpaired *t* test, *P* = 0.041).

**Figure 2:**
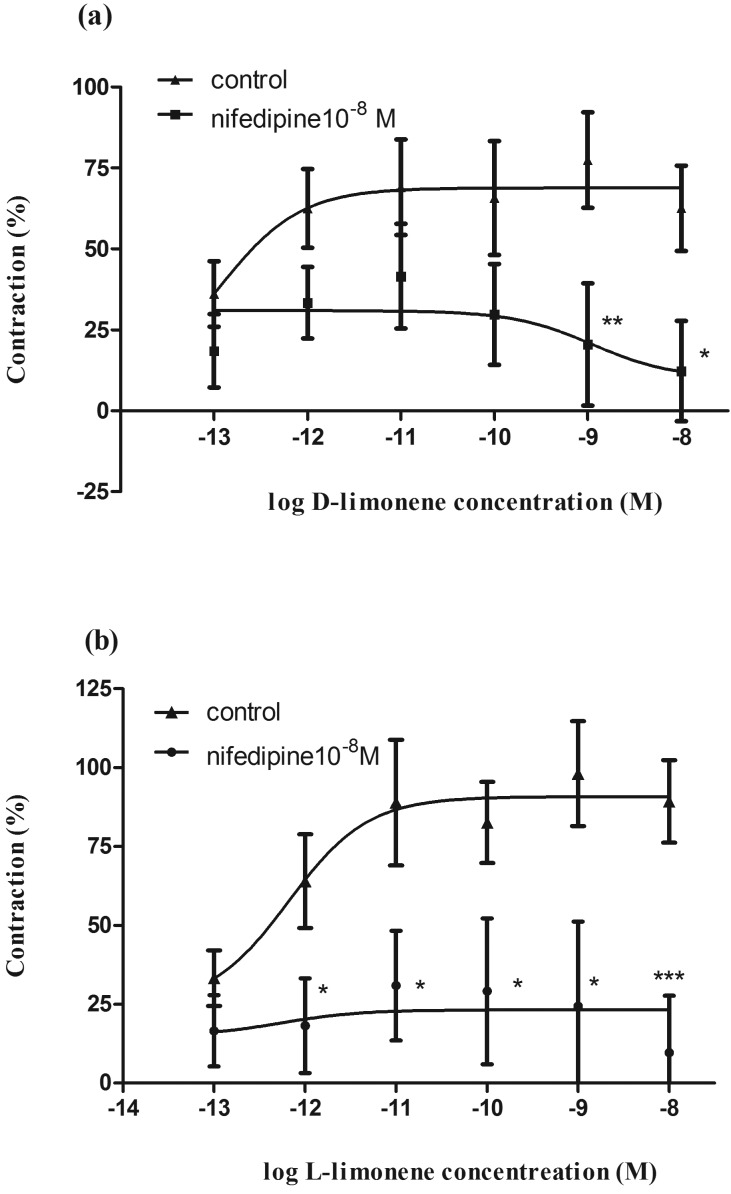
The effect of d-limonene (**A**) and l-limonene (both 10^−13^-10^−8^ M) (**B**) on uterine contraction after nifedipine (10^−8^ M) pre-treatment. Change in contraction was calculated using the areas under the curve and expressed in percent ± standard deviation (unpaired *t* test, *P* = 0.048).

Both d- and l-limonene caused myometrial contraction in a dose-dependent manner (Figure 1). L-limonene caused significantly stronger myometrial contraction (Emax = 104.1% ± 35.33%) (*P* = 0.035) than d- limonene (Emax = 75.3% ± 34.4%). Pre-treatment with nifedipine (10^−8^ M) (Figure 2A) significantly decreased the myometrial contracting effect of both d-limonene (Emax = 9.9% ± 37.4%) (*P* = 0.009) and l-limonene (Figure 2B) (Emax = 23.21% ± 28.3%) (*P* < 0.001).

**Figure 3:**
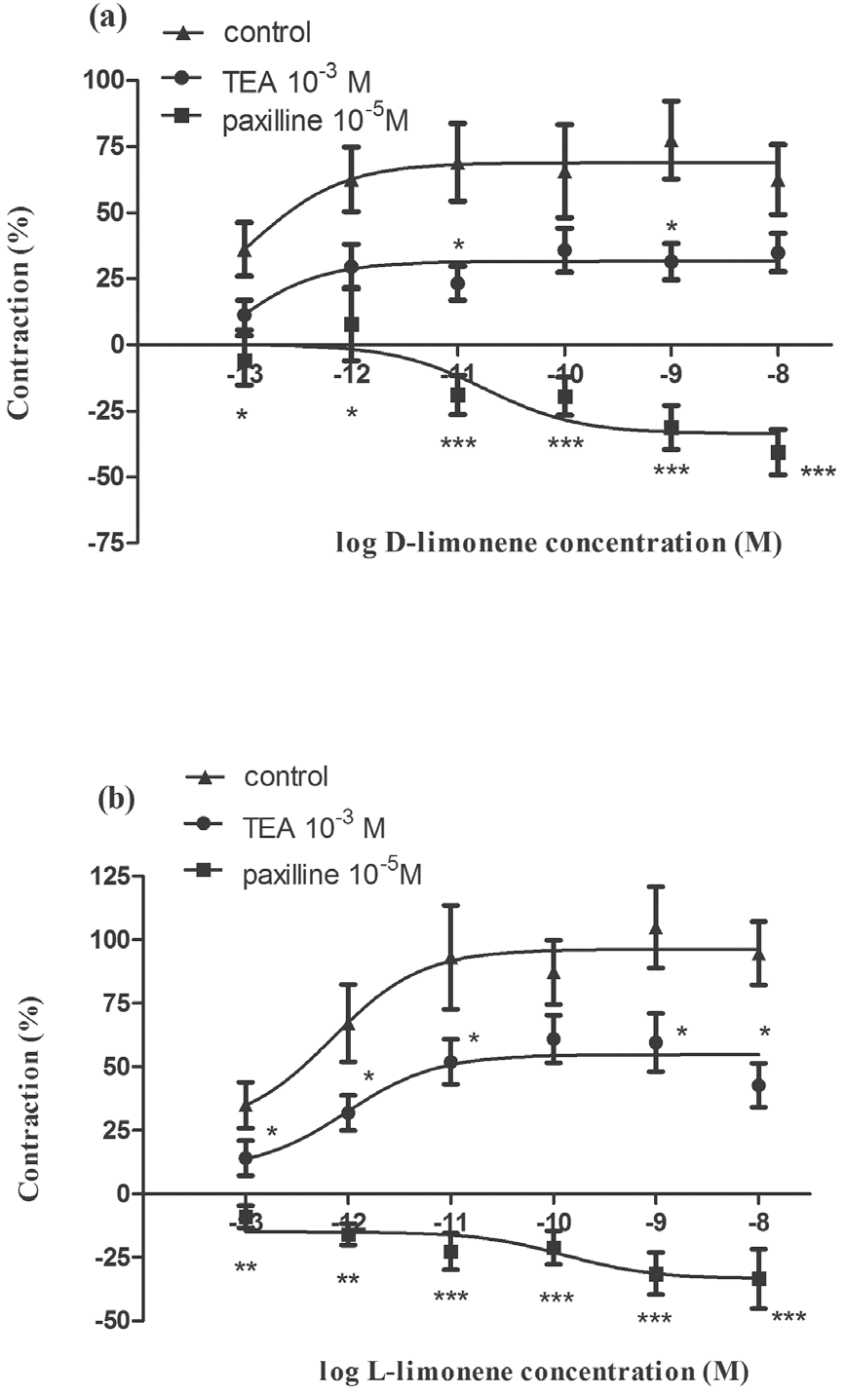
The effect tof d-limonene (**A**) and l-limonene (both 10^−13^-10^−8^ M) (**B**) on uterine contraction after tetraethyl-ammonium (TEA) (10^−3^ M) or paxilline (10^−5^ M) pre-treatment. Change in contraction was calculated using the areas under the curve and expressed in percent ± standard deviation (unpaired *t* test, *P* = 0.050).

Pre-treatment with TEA (10^−3^ M) (Figure 3A) significantly decreased the myometrial contracting effect of both d-limonene (Emax = 31.6% ± 21.88%) and l-limonene (Emax = 54.6% ± 31.23%) (*P* < 0.049), while paxilline (10^−5^ M) reversed this action and caused muscle relaxation (Emax = -33.5% ± 27.02% for d-limonene, *P* < 0.001), (Emax = -33.2% ± 26.84% for l-limonene, *P* < 0.001).

**Figure 4:**
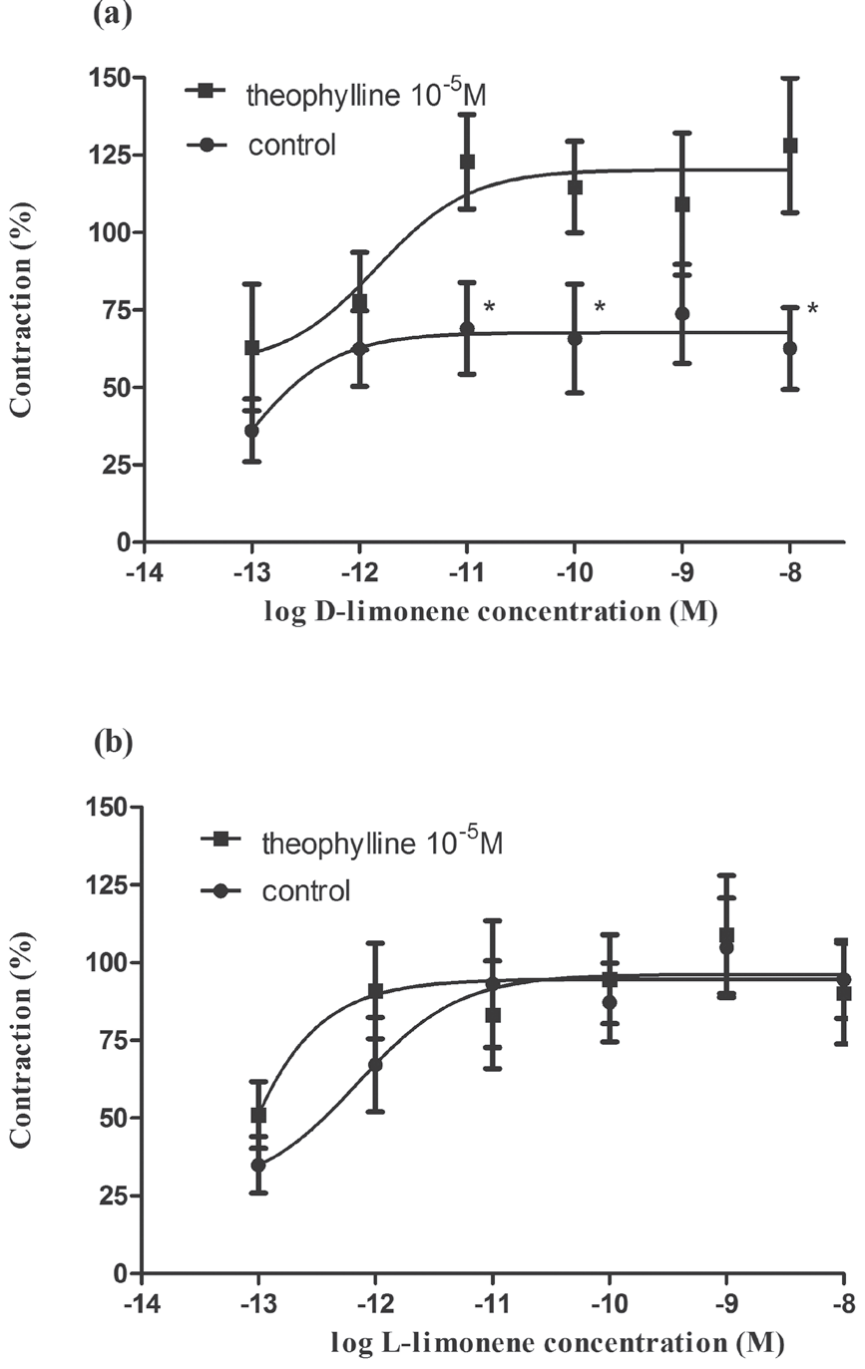
The effect of d-limonene **(A)** and l-limonene (both 10^−13^-10^−8^ M) **(B)** on uterine contraction after theophylline (10^−5^ M) pre-treatment. Change in contraction was calculated using the areas under the curve and expressed in percent ± standard deviation (unpaired *t* test, *P* = 0.048).

Pre-treatment with theophylline (10^−5^M) significantly decreased the myometrial contracting effect of d-limonene (Emax = 75.34% ± 51.28%) (*P* = 0.048) (Figure 4A), but did not alter the contracting effect of l-limonene (Emax = 94.6% ± 25.95%) (Figure 4B).

### Measurement of preventive effect of limonene against MGO-induced oxidative stress

**Figure 5:**
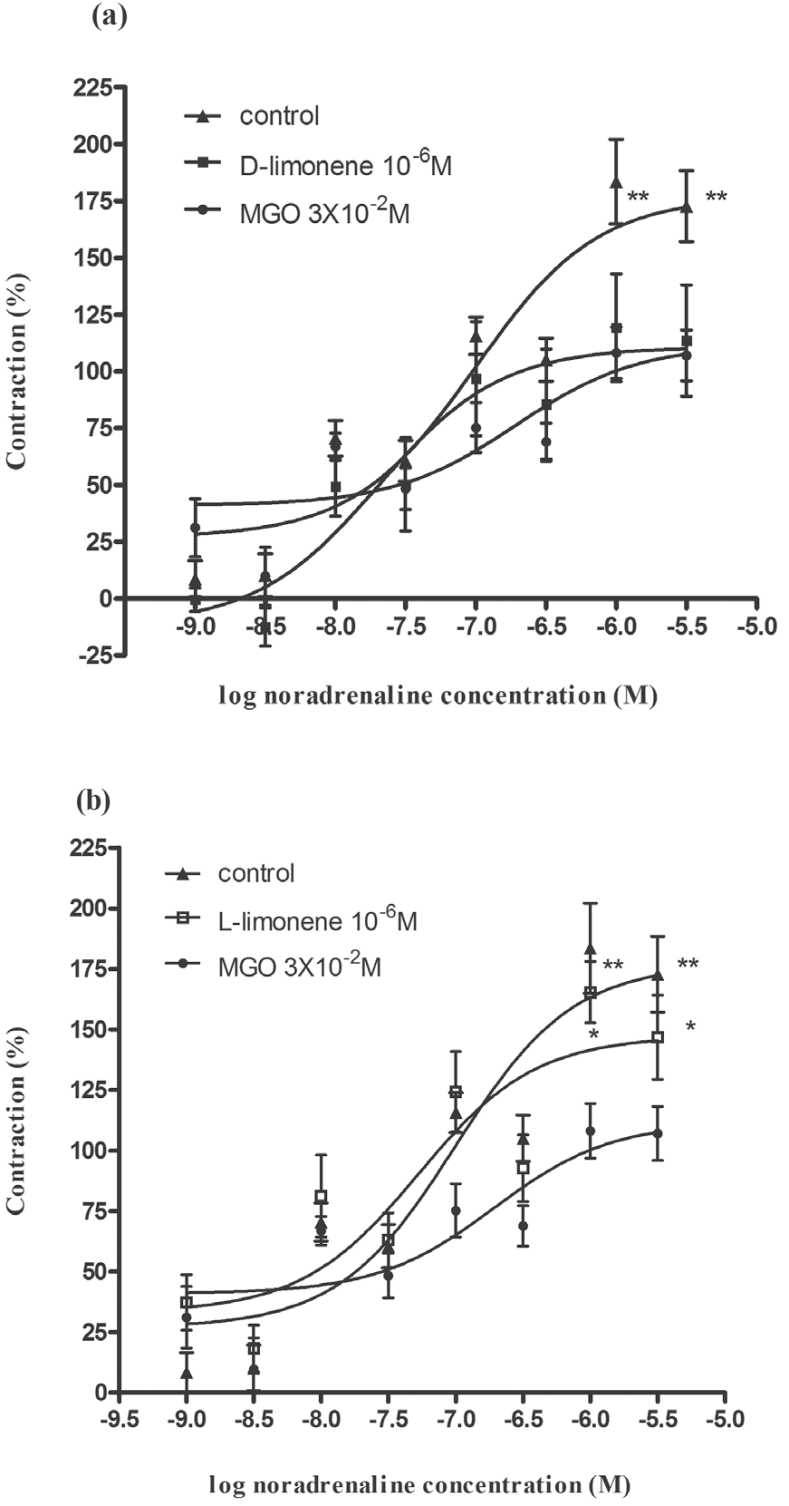
The effect of d-limonene (10^−6^ M) **(A)** and l-limonene (10^−6^ M) **(B)** on the noradrenaline-evoked (control) uterine contractions in the presence of methylglyoxal (3 × 10^−2^ M) and β-adrenoreceptor antagonist propranolol (10^−5^ M) (Dunnett’s multiple comparison test, *P* = 0.043).

**Figure 6:**
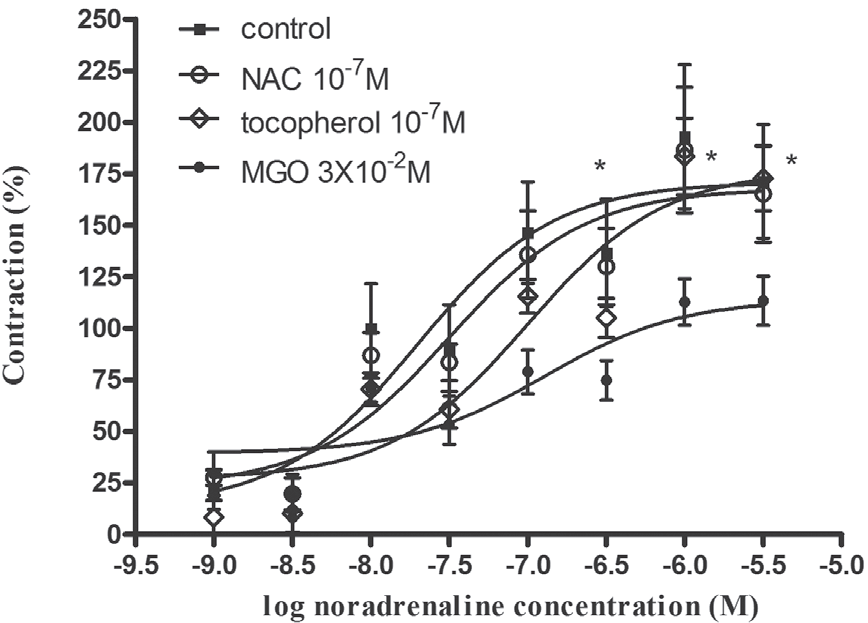
The effect of N-acetylcysteine (10^−7^ M) and tocopherol (10^−7^ M) on the noradrenaline-evoked (control) uterine contractions in the presence of methylglyoxal (3 × 10^−2^ M) and β-adrenoreceptor antagonist propranolol (10^−5^ M) (Dunnett's multiple comparison test, *P* = 0.025).

MGO treatment (3 × 10^−2^ M) significantly reduced the NA-evoked smooth muscle contraction *in vitro* (*P* = 0.003). While d- limonene (10^−6^ M) was ineffective (Figure 5A), l-limonene (10^−6^ M) significantly decreased the effect of MGO (*P* = 0.043) (Figure 5B). Both NAC and tocopherol significantly decreased the effect of MGO on myometrium (*P* = 0.003) (Figure 6).

### cAMP experiments

**Figure 7:**
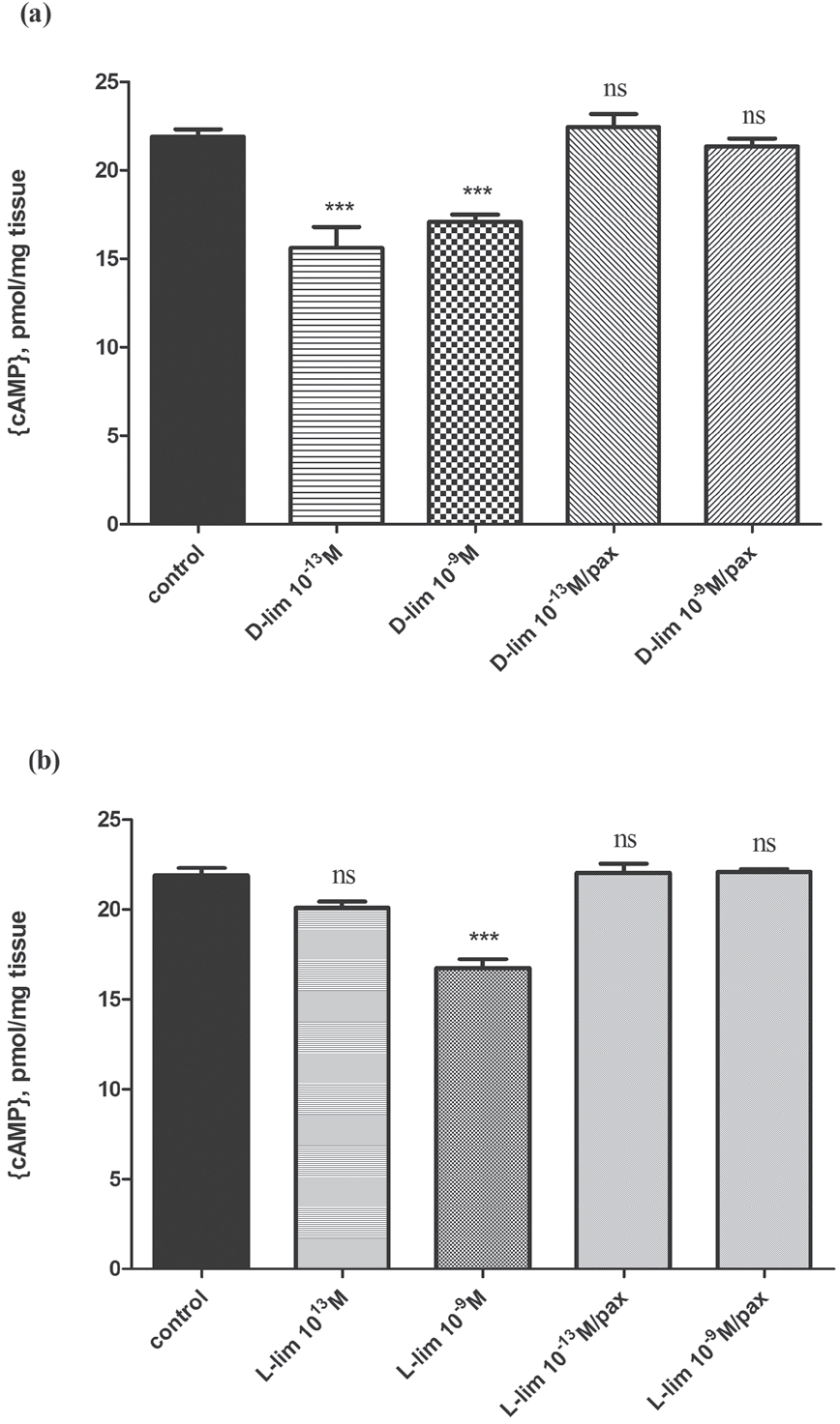
The effect of d-limonene (**A**) and l-limonene (**B**) on the myometrial cyclic adenosine monophosphate (pmol/mg tissue ± standard deviation) in the presence of isobuthylmethylxantine (10^−3^ M), forskolin (10^−5^ M) (control), and paxilline (10^−5^ M) (ANOVA followed by Dunnett's multiple comparison test, *P* < 0.001).

In two different concentrations (10^−13^, 10^−9^ M) d-limonene decreased cAMP synthesis (Figure 7A). Paxilline (10^−5^ M) pre-treatment prevented the effect of d-limonene on cAMP production, however it did not cause any significant changes compared to controls (IBMX, forskolin, NA).

L-limonene significantly decreased the cAMP level at the concentration of 10^−9^ M, but at 10^−13^ M concentration exhibited no significant effects compared to the control. Paxilline (10^−5^ M) pre-treatment prevented the effect of l-limonene on cAMP production, but did not change the cAMP level compared to controls (Figure 7B).

## Discussion

Our study showed that both d- and l-limonene caused myometrial contraction in a dose-dependent manner. The myometrial effect of limonene was decreased by pre-treatment with nifedipine and TEA, while in the presence of paxilline, limonene did not cause myometrial contraction. These findings suggest that increased intracellular calcium pathways and BK_Ca_ channels can be involved in the action mechanism of limonene.

One study (28) showed that limonene was an adenosine A_2A_ receptor agonist. A_2A_ receptor agonists bind to G-coupled receptors and stimulate protein kinase A and induce formation of inositol 1,4,5-triphosphate, which stimulates opening of endoplasmic reticulum Ca^2+^ channels, and results in increased intracellular Ca^2+^ levels (29). We investigated the effect of limonene on adenosine receptors in the presence of theophylline. Theophylline pre-treatment significantly decreased the myometrial contracting effect of d-limonene, but did not affect the myometrial contracting effect of l-limonene. There are two main sources of intracellular Ca^2+^ level increase in the myometrium: voltage-gated L-type Ca^2+^ channel influx and sarcoplasmic reticulum (SR) release (30). The L-type Ca^2+^ channel activity is also regulated by the SR (31), and its depletion activates the L-type Ca^2+^ channel. The results of the current study are in accordance with these findings and suggest that d-limonene stimulates A_2A_ receptors, which causes opening of L-type Ca^2+^ channels and hence causes myometrial contraction, while l-limonene causes myometrial smooth muscle contraction independently of A_2A_ receptors.

To find an explanation for this, we measured the uterine cAMP accumulation since cAMP levels are crucial in the control of smooth muscle contraction and relaxation. Phosphodiesterase inhibitor IBMX was used to block the degradation of the generated intracellular cAMP, while forskolin was used to enhance the activity of adenyl cyclase (18). D- and l-limonene decreased the cAMP level, which contributed to their myometrial contracting effect. After paxilline pre-treatment, limonene did not alter the cAMP level compared to the control. BK_Ca_ channels are important in maintaining myometrial quiescence (32). They are activated by increased intracellular Ca^2+^ levels and counteract depolarizing effects of Ca^2+^ by deactivating voltage-gated calcium channels or through increased transport activity of Na^+^/Ca^2+^ exchangers (33). It is plausible that paxilline-induced BK_Ca_ channel blocking inhibits intracellular calcium sparks, which may explain why d- and l-limonene had weaker contracting effect on the myometrium. Additionally, TEA slightly decreased the myometrial contracting effect of limonene, which corresponds to the fact that TEA just partially blocks the BK_Ca_ channels (34). The increased intracellular Ca^2+^ level directly inhibits the cAMP system (35), therefore, if the BK_Ca_ channel is blocked and there is weaker calcium flow, cAMP level can return to normal.

Results of several studies suggest the terpenoids may have antioxidant effects (13,36). Therefore, we investigated the protective effect of limonene on oxidative damage of uterine contractility in pregnancy. MGO is a reactive alpha-dicarbonyl compound synthesized in various biochemical processes in most mammalian cells, including vascular endothelial cells and smooth muscles (37). It increases superoxide production and inhibits NA-induced smooth muscle contraction in rat isolated carotid artery, which can be prevented by NAC pre-treatment (19). First we investigated if the same model can be applied to investigating myometrial smooth muscle contraction and relaxation. MGO decreased the NA-induced myometrial smooth muscle contraction, which was prevented by NAC and tocopherol pre-treatment. L-limonene significantly decreased the inhibitory effect of MGO, although this effect was weaker than that of NAC and tocopherol. This study provides the first evidence that limonene in low concentrations causes myometrial smooth muscle contraction. It activates A_2A_ receptors and mediates voltage-gated Ca^2+^ channel opening, which is the major mechanism of Ca^2+^ for myometrial smooth muscle contraction. We also demonstrated for the first time that MGO treatment has inhibitory effects on the NA-evoked myometrial smooth muscle contraction, which could be prevented by l-limonene, NAC, and tocopherol pre-treatment. Therefore, l-limonene has a contraction maintaining effect on the pregnant uterus. Although d-limonene is the dominant enantiomer in most plants, both enantiomers are used as specific blends in different consumer products (38).

A limitation of our study is that we did not carry out contractility studies on human myometrium, therefore we can conclude that the same effect can be obtained in humans. However, our present findings suggest that limonene-containing products may increase uterine contractions during pregnancy.
